# Sustainable textiles through microbe-produced bioleather

**DOI:** 10.1038/s44172-022-00022-7

**Published:** 2022-09-16

**Authors:** Miranda Vinay

**Affiliations:** Communications Engineering, https://www.nature.com/commseng/

**Keywords:** Sustainability

## Abstract

A recent publication in *Environmental Sciences*: Advances reports flame-retardant, colored and soil-biodegradable nanocellulose bioleather with tensile strength and ductility competitive with traditional leather. Further, the researchers report that these microbial biotextiles have a thousandfold reduction in human toxicity levels compared to cow leather with a carbon footprint lower than cow leather, synthetic leather and cotton.

The textile industry is due for a green overhaul. Without serious renovation, it will likely be responsible for 25% of the global carbon budget by 2050^1^. Plant-based synthetic leathers are a hopeful lifeline to this industry. But, similarly to traditional leathers, they use harsh chemical treatments to stabilize and strengthen the materials and color them for textile applications. These processes compromise material biodegradability and introduce human and ecological toxicity.

**Figure:**
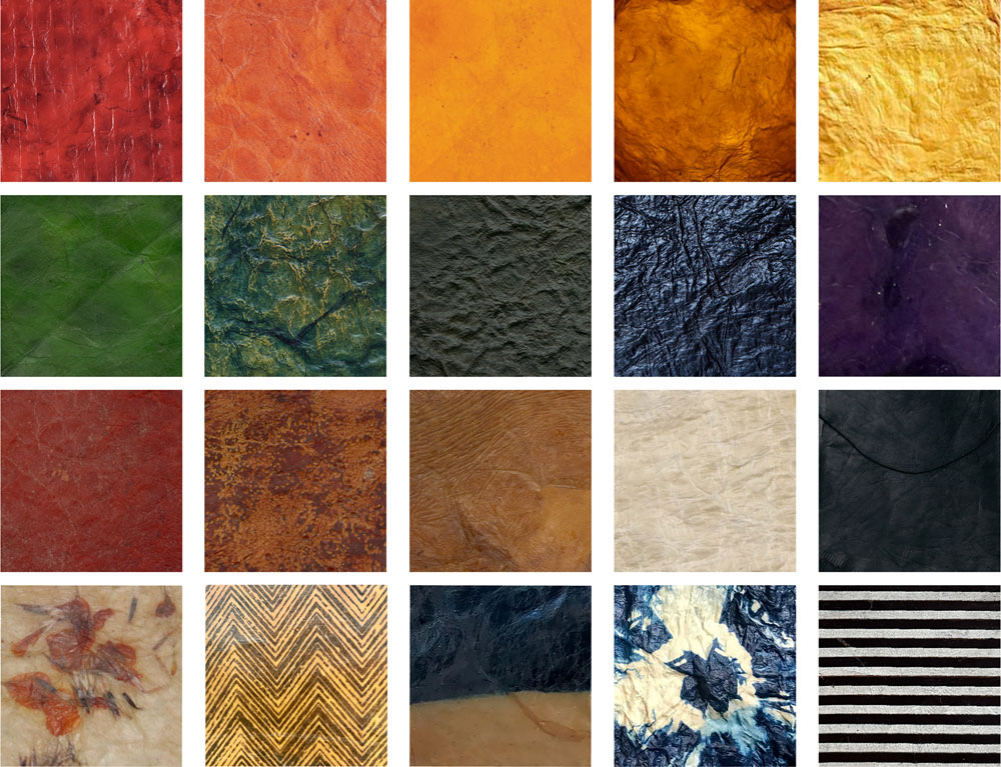
Theanne N. Schiros

Dr. Theanne N. Schiros and collaborators, including Romare Antrobus and Dr. Helen H. Lu, harnessed the bacteria *Acetobacter xylinus* with saccharomyces to produce nanocellulose, a highly crystalline biopolymer, which can self-assemble into layers forming a fabric. Then, they looked to the past for making more sustainable tanning processes for strengthening and stabilization. Leather is an ancient material. Past civilizations tanned leather with smoke and brain matter via methods long since lost to history, but suggested to be forms of aldehyde tanning^2^. Aldehydes from the tanning agents toughen the textile by covalently crosslinking amines within the collagen molecules from the leather matrix^3^. The researchers postulated that the amphiphilic lecithin abundant in brain tissue stabilizes the tanning emulsions and facilitates the interaction between the oils and collagen, perhaps also interacting with the abundant hydroxyl groups of cellulose. For more sustainable tanning, Dr. Schiros replaced brain lecithin with an emulsion of plant-based lecithin (a common by-product of the edible oil industry), sunflower oil and water. The researchers probed four tanning methods for these bacteria-produced bioleathers; without treatment, tanned with lecithin, smoked, and treated with a combination of lecithin and smoking to explore their practical potential.

Dr. Schiros and collaborators found that lecithin-tanned materials showed higher tensile strength as compared to traditional leather for up to 33% strain. The new materials were also incombustible, another important property of textiles. Further, a cradle-to-gate life cycle assessment showed a lower carbon footprint and a thousandfold reduction in human toxicity as compared to cow leather modeled as a by-product of the livestock industry. Not surprisingly, the carbon footprint is 100 times lower than leather modeled as co-product. When compared to synthetic leather made from polyurethane-coated polyester and cotton, the carbon footprint is reduced by 96.8% and 94.3%, respectively. And the lecithin-treated bioleather visibly deteriorated and crumbled easily after 60 days of being buried in soil. Dr. Schiros also showed that these “leathers” can make colorful shoes and wallets, using natural dyes in the bacteria-growing medium thus eliminating wasteful dip-dyeing production steps.

Dr. Schiros and her team explored the mechanisms underlying the mechanical effects using atomic- and molecular-level chemical analysis. The results suggested that the stabilization and improvement of mechanical properties were the result of a disruption in hydrogen bonding physical crosslinks, and an increased interaction between cellulose chains that more effectively transferred and distributed applied stress. As a flame-retardant, they believe higher phosphorylation was responsible for forming char rather than combusting. The lecithin tanning resulted in a nearly four-fold increase in phosphorous content than the control. Additionally, substituting natural alternatives to harsh, carcinogenic chromium VI derivatives drove the reduction in human toxicity when compared to tanning cow leather.

When asked about the impact of this work, Dr. Schiros wrote “The biofabrication approach developed here represents a conduit for improved biomaterials and processes at scale, aligned with the defining principles and practices of green chemistry, including: less hazardous syntheses, design for degradation, integration of material and energy flows, and inherently safer chemistry, to simultaneously meet environmental and global development goals.”

The discovery, methodology and techniques developed also have impact for cultural heritage research, noted Dr. Schiros. The team is now collaborating with scientists at the Metropolitan Museum of Art in New York City to develop a conservation studies data base for artifacts in their cultural heritage collections and to understand the mechanism behind historic brain and organ tanning.

The original article can be found here: Schiros, T. et al. Microbial nanocellulose biotextiles for a circular materials economy. Environmental Science: Advances 284, (2022); 10.1039/d2va00050d.
