# Author Correction: Sustainable textiles through microbe-produced bioleather

**DOI:** 10.1038/s44172-022-00040-5

**Published:** 2022-11-28

**Authors:** Miranda Vinay

**Affiliations:** Communications Engineering, https://www.nature.com/commseng/

**Keywords:** Sustainability

Correction to: *Communications Engineering* 10.1038/s44172-022-00022-7, published online 16 September 2022.

The final sentence of the body text was omitted from this article. It should have read “The original research article can be found here: Schiros, T. et al. Microbial nanocellulose biotextiles for a circular materials economy. Environmental Science: Advances 284, (2022); 10.1039/d2va00050d”. The original article has been corrected.

